# Gut-brain connections in neurodegenerative disease: immunotherapeutic targeting of Bin1 in inflammatory bowel disease and Alzheimer’s disease

**DOI:** 10.3389/fphar.2023.1183932

**Published:** 2023-07-13

**Authors:** Sunil Thomas, George C. Prendergast

**Affiliations:** Lankenau Institute for Medical Research, Wynnewood, PA, United States

**Keywords:** Alzheimer’s disease, gut-brain connection, immunotherapy, BIN1, inflammatory bowel disease

## Abstract

Longer lifespan produces risks of age-associated neurodegenerative disorders such as Alzheimer’s disease (AD), which is characterized by declines in memory and cognitive function. The pathogenic causes of AD are thought to reflect a progressive aggregation in the brain of amyloid plaques composed of beta-amyloid (Aß) peptides and neurofibrillary tangles composed of phosphorylated tau protein. Recently, long-standing investigations of the Aß disease hypothesis gained support via a passive immunotherapy targeting soluble Aß protein. Tau-targeting approaches using antibodies are also being pursued as a therapeutic approach to AD. In genome-wide association studies, the disease modifier gene Bin1 has been identified as a top risk factor for late-onset AD in human populations, with recent studies suggesting that Bin1 binds tau and influences its extracellular deposition. Interestingly, before AD emerges in the brain, tau levels rise in the colon, where Bin1—a modifier of tissue barrier function and inflammation—acts to promote inflammatory bowel disease (IBD). This connection is provocative given clinical evidence of gut-brain communication in age-associated neurodegenerative disorders, including AD. In this review, we discuss a Bin1-targeting passive immunotherapy developed in our laboratory to treat IBD that may offer a strategy to indirectly reduce tau deposition and limit AD onset or progression.

## Introduction

As average life expectancy has increased since the 19th century, the prevalence of aging-associated diseases including dementia has also increased. Among the various types of dementia, late-onset Alzheimer’s disease (LOAD) is the most common form in late life. Alzheimer’s disease (AD) is a progressive neurodegenerative disorder of the brain that destroys memory, cognitive skills, spatial orientation, and deterioration of intellectual capacity needed to perform the simplest tasks. AD is believed to be caused by the accumulation of amyloid plaques and neurofibrillary tangles in the brain, disrupting its functions. The incidence and prevalence of AD continue to rise as global populations age (Cummings et al., 2016). Globally, there are >55 million people living with dementia in 2020, with expectations that this number will double every 20 years to 78 million in 2030 and >140 million in 2050 (https://www.alz.co.uk/research/statistics). Currently, AD causes estimated economic losses of US$ 321 billion worldwide with costs expected to grow to US$ 1 trillion by 2050 (www.alz.org). As yet there is no cure for AD and effective early diagnosis is also lacking (Qin et al., 2009).

In 1906, psychiatrist and neuropathologist Alois Alzheimer pioneered histopathological studies of this devastating disease with pivotal observations of distinctive plaques and neurofibrillary tangles in the brain histology of a woman with dementia (Hippius and Neundörfer, 2003). Many years later, these structures were defined as aggregated Aβ peptides and hyperphosphorylated tau, respectively, as defining pathological features of AD (Liu et al., 2004; Saha and Sen, 2019). Overproduction and accumulation of these abnormal proteins in the brain disrupt neuronal function and may ultimately lead to neuronal death and cognitive decline. However, AD is characterized by additional pathological changes in the postmortem brain, including synaptic loss, oxidative damage, and activated inflammatory cells, in addition to amyloid plaques and neurofibrillary tangles (Medeiros et al., 2011). Cytokines in the tumor necrosis factor (TNF) superfamily appear to promote accelerated cell death rates in neurodegenerative processes. Among these, the pro-apoptotic/pro-inflammatory cytokine Tumor necrosis factor-Related Apoptosis-Inducing Ligand (TRAIL) is expressed in the brain of AD patients but is completely absent in the brain of non-demented patients (Uberti et al., 2004). TRAIL has been found to be a potent driver of neuronal loss in both chronic and acute neurodegenerative processes, including those related to Aβ accumulation (Burgaletto et al., 2020).

Neuroimaging techniques may aid in diagnosing AD, helping distinguish it from other causes of dementia (Cummings, 2004). Biomarkers developed for AD neuroimaging techniques have helped illuminate pathophysiological mechanisms such as structural and functional decline, white matter decline, and pathology aggregation, leading to improved diagnosis and treatment aggregation (Marquez and Yassa, 2019). Notably, both clinical investigations and preclinical model studies suggest that pathophysiological features of AD may arise years before the onset of neurological symptoms, highlighting needs for early detection and intervention in disease management (Therriault et al., 2022a; Therriault et al., 2022b).

## Tau in Alzheimer’s disease

Tau is a microtubule-associated protein that has critical physiological roles in microtubule assembly and stability. Microtubules are dynamic polymers composed of alpha and beta tubulins that form a hollow tube-like structure. In neurons, they play essential roles in cell division, shape, motility and intracellular transport, and tau binding is vital for regulating microtubule assembly and disassembly in these processes. In normal cells, phosphorylation of tau regulates its binding to microtubules. In AD, tau is hyperphosphorylated such that it can no longer bind or stabilize microtubules. Instead, hyperphosphorylated tau accumulates in neurofibrillary tangles (NFT), a chief hallmark of AD histopathology, fomenting neuronal damage and cell death (Grundke-Iqbal et al., 1986). Loss of the microtubule stabilizing function of tau also disrupts axonal transport processes, further promoting to neuronal dysfunction and degeneration (Brion et al., 1985; Wood and Zinsmeister, 1989; Barbier et al., 2019; Saha and Sen, 2019; d’Errico and Meyer-Luehmann, 2020). This type of tau dysfunction occurs not only in AD but also other neurodegenerative disorders, now collectively referred to as tauopathies (Chung et al., 2021).

Neurofibrillary tangles (NFT) containing hyperphosphorylated tau have a characteristic six-stage brain distribution pattern (Braak and Braak, 1991). In stages I and II, NFT are limited to the transentorhinal region of the brain; in stages III and IV, NFT are also found in limbic regions, including the hippocampus; and in stages V and VI, NFT are distributed widely in neocortical regions. In considering this distribution pattern, recent studies have suggested that tau pathology may spread between connected regions in the brain in a “prion-like” manner (Mudher et al., 2017). How this occurs is obscure, but might involve transmission along neuroanatomically connected regions to drive the progressive and widespread neuronal dysfunction that is observed in tauopathies (Zhang Y. et al., 2021).

Tau expression and molecular structure in the brain is well characterized. In normal adult brain, six distinct tau isoforms are generated from a single tau gene by alternative RNA splicing. All isoforms can be phosphorylated at sites that regulate microtubule binding. In the AD brain, these same isoforms are hyperphosphorylated at ∼30 reported sites, only some of which are phosphorylated to any significant extent in normal brain. Overall, tau expressed in AD brain contains up to 4-fold more phosphate/mole protein, ablating the normal function of tau in stabilizing microtubules while seeding formation of aberrent tau aggregates in NFT (Liu et al., 2004). In its normal form, tau is also modified by protein O-glycosylation at multiple sites, through addition of the monosaccharide β-N-acetylglucosamine (GlcNAc) to serine/threonine residues via an O-linked glycosidic bond. Notably, these modifications negatively regulate tau phosphorylation in a site-specific manner, with decreased O-glycosylation, an apparent essential cause of tau hyperphosphorylation in AD and other tauopathies (perhaps influenced by deficient brain glucose metabolism, a common feature of AD and other tauopathies) (Liu et al., 2004).

Notably, genetic evidence suggests that in the absence of amyloid-beta (Aβ) plaques aberrant tau isoforms are sufficient to trigger neurodegeneration (Medeiros et al., 2011). On this basis, limiting the accumulation and spreading of tau aggregates may offer a distinct approach or cooperative approach (e.g., with anti-Aß antibodies) to limit development and progression of AD and other tauopathies. Present knowledge suggests that restoring tau O-glycosylation may offer an approach in principle, although tractability is uncertain. Alternately, attacking the underlying mechanisms of tau deposition, aggregation or propagation may offer therapeutic strategies to treat tauopathies like AD (Medeiros et al., 2011).

## Genetics and function of Bin1

Bin1 is a highly conserved gene in evolution that encodes membrane-associated and nuclear functions distinguished by complex patterns of tissue-specific alternate RNA splicing and protein-protein binding. Genetic and biochemical studies in yeast, flies and rodents have implicated Bin1 orthologs in diverse cellular processes, including membrane bending and membrane-actin dynamics, cell polarity, tissue barrier function, endocytosis, apoptosis, DNA repair and inflammatory signaling (Prendergast et al., 2009). Bin1 is the prototype member of the Bin1/Amphiphysin/RVS167 (BAR) gene family named for the structurally distinct BAR domain. Bin1 is expressed in all cells, but most highly as tissue-specific isoforms in muscle and brain (Prendergast et al., 2009).

The human BIN1 gene encompasses 20 exons, encoding an N-terminal BAR domain (exons 1–10), muscle-specific phosphoinositide-binding motif (exon 11), brain-specific clathrin and AP2 binding domain (CLAP; exons 12–16), MYC-binding domain (MBD; exons 17–18) and SH3 domain (exons 19–20). The BAR domain, included in all splice isoforms, encodes a canonical function in membrane bending/shaping and an alternate ‘moonlighting’ function in Set1/RAD6-mediated transcriptional repression (Prendergast et al., 2009). The alternately spliced CLAP domain exons that encode functions in endosome trafficking and synaptic vesicle recycling are included in brain isoforms and tumor-specific isoforms. The alternately spliced MBD is implicated in binding Myc transcription factors and regulating histone acetylation. Lastly, the SH3 domain, included in all splice isoforms, binds numerous regulatory and structural proteins that help determine localization of Bin1 proteins, including tau (Taga et al., 2020).

## The role of Bin1 in Alzheimer’s disease

**FIGURE 1:**
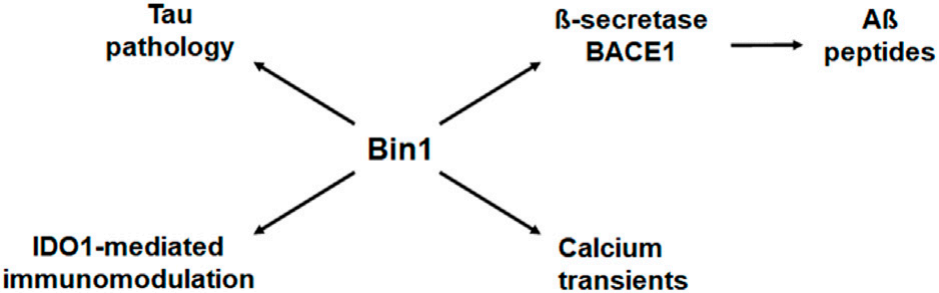
Bin1 modifies multiple disease processes in Alzheimer’s disease. Bin1 interacts with and influences the deposition of tau, but it also impacts production of Aß peptides, local immune modulation, and calcium homeostasis in the setting of AD (Tan et al., 2013). Bin1 interacts with ß-secretase BACE1 to influence expression of Aß peptides (Miyagawa et al., 2016). Bin1 expression also influences adaptive immunity through its regulation of indoleamine 2,3-dioxgenase (IDO), and innate immunity through IDO-mediated control of inflammatory cytokines, most prominently IL-6 (Muller et al., 2023). Lastly, Bin1 has also been implicated in the control of calcium transients that exert pleiotropic cellular responses. Thus, the effects of Bin1 on tau may be accompanied by additional effects in a deeper disease context to modify AD progression.

Multiple Genome-Wide Association Studies (GWAS) have identified an overlapping set of genetic variants associated with AD in diverse human populations, including with sporadic late-onset AD (LOAD) as the most common type. In particular, genetic polymorphisms associated with LOAD have been identified in human genes that function in endosome recycling, including BIN1, CLU, PICALM, RIN3, and CD2AP. Endosome recycling is crucial for protein sorting and trafficking events that maintain cellular homeostasis and polarity (Seshadri et al., 2010; Crotti et al., 2019; Kunkle et al., 2019). Of these genes, BIN1 exhibits the strongest association with AD. Interestingly, the association between BIN1 and AD appears independent of the established association between the cholesterol transport protein Apolipoprotein E (APOE) and AD. Overall, BIN1 has been argued to be the first or second most important genetic risk factor for LOAD (Cruz-Sanabria et al., 2021). Figure 1 summarizes various functional impacts that Bin1 may conceivably exert in AD, based on present knowledge.

Physical binding of tau to Bin1 has been implicated in AD pathogenesis. The proline-rich domain (PRD) of tau mediates binding to Bin1. In its PRD, tau contains seven PXXP motifs (Usardi et al., 2011) that directly bind to the Bin1 SH3 domain in a phosphorylation-dependent manner (Chapuis et al., 2013; Sottejeau et al., 2015; Malki et al., 2017; Sartori et al., 2019). Notably, tau phosphorylation at Thr231 and other nearby residues negates Bin1 SH3 binding, precluding normal binding and enabling accumulation and aggregation of hyperphosphorylated tau that causes AD pathophysiology (Sottejeau et al., 2015). Consistent with a role for Bin1 in driving tau pathology, Bin1 has been reported to colocalize with NFT-containing neurons (Holler et al., 2014) where it is associated with elevated tau phosphorylation levels (Wang et al., 2016).

Recent functional studies in the PS19 mouse model of tauopathy offer additional genetic support of a role for Bin1 in promoting tau-dependent AD (Crotti et al., 2019). First, Bin1 overexpression promoted vesicle-mediated secretion of tau in the brain, exacerbating tau pathology during disease progression in the model. Second, tissue-specific genetic deletion of Bin1 from microglia reduced tau secretion and decreased tau spreading in the brain. Third, Bin1 ablation in microglia also reduced expression of heat-shock proteins that have been implicated in tau proteostasis previously (male-specific effect) (Crotti et al., 2019). Taken together, these findings corroborate the hypothesis that Bin1 expression in microglia contributes to AD progression by promoting deposition of tau synthesized in microglia into the brain extracellular matrix.

Other studies in rodent models of AD support a functional role for Bin1 in modulating tau pathology. Bin1 overexpression was reported to cause microstructural changes in murine hippocampal circuits (Daudin et al., 2018), a region of the brain that exhibits tau pathology relatively early in clinical AD. In primary rat neuron cultures, Bin1 knockdown led to accumulation of phosphorylated tau at synapses, altering synapse structure, disrupting tau secretion and promoting phosphorylated tau-mediated synaptotoxicity (Glennon et al., 2020). These findings parallel earlier evidence that Bin1 may affect AD development by modulating tau effects at synapses, possibly including synaptic activity-dependent tau release (Pooler et al., 2013).

Studies in the genetically malleable fruit fly *Drosophila* encourage the concept that Bin1 supports tau pathology. Whereas yeast and mammals encode two evolutionarily conserved BAR family genes, Bin1 and Bin3, flies encode only a single Bin1 ortholog (this gene is confusingly termed *Amphiphysin [Amph]*, despite its homology to mammalian Bin1 rather than the mammalian gene termed amphiphysin/AMPHI). In one study, overexpression of the fly Bin1 ortholog *Amph* was observed to increase tau neurotoxicity in flies (Chapuis et al., 2013). In a second study, *Amph* knockdown was observed to promote tau propagation between neurons (Calafate et al., 2016). This observation was suggested to be relevant to familial AD, where Bin1 levels were reported to be reduced rather than elevated, as the case in sporadic LOAD (Perdigão et al., 2021). In another study employing a fly model of tauopathy, *Amph* knockdown was observed to reduce tau-induced actin inclusions (Drager et al., 2017), a feature of tau pathophysiology, consistent with evidence that Bin1 exerts actin bundling and filament stabilizing effects (Prendergast et al., 2009) that are altered in various neurological disorders, including AD. While further research is needed to fully illuminate the mechanisms by which Bin1 affects tau pathophysiology and its role in the pathogenesis of AD, functional studies to date reinforce the hypothesis Bin1 influences AD pathogenesis via tau pathology and synaptic dysfunction.

In addition to tau, AD is characterized by the extracellular plaque deposits of β-amyloid peptide (Aβ) (Murphy and LeVine, 2010). Beta-site amyloid precursor protein cleaving enzyme 1 (BACE1) is a type 1 transmembrane aspartyl protease expressed predominantly in neurons of the brain and responsible for the production of Aβ. Depletion of Bin1 increases cellular BACE1 levels through impaired endosomal trafficking and reduces BACE1 lysosomal degradation, resulting in increased Aβ production (Miyagawa et al., 2016).

Interestingly, Aβ is considered an antimicrobial peptide. Insoluble Aβ oligomers bind to microbial cell wall carbohydrates via a heparin-binding domain (Kumar et al., 2016). According to the “infection hypothesis” the antimicrobial peptide Aβ is produced against bacteria, fungi, and viruses. The production of Aβ as an anti-microbial peptide (AMP) will be beneficial on first microbial challenge but will become progressively detrimental as the infection becomes chronic and reactivates from time to time. The capacity of the host to remove excess Aβ decrease over time due to microglial senescence and microbial biofilm formation. The biofilm aggregates with Aβ to form the plaques in the brain of AD patients (Fulop et al., 2018).

## Bin1 in inflammatory bowel disease

Our team at Lankenau pioneered genetic studies of Bin1 in the mouse by creating the initial complete, mosaic and tissue-specific deletion strains broadly employed in the field. By this route, we accumulated evidence that Bin1 acts as a modifier of disease severity in many settings, including cancer, cardiac arrhythmia, heart failure, inflammatory bowel disease (IBD) and neurodegenerative disease (Muller et al., 2003; 2004; Chang et al., 2007a; 2007b; 2012; Laury-Kleintop et al., 2015; Miyakawa et al., 2016). Complete deletion of Bin1 causes perinatal lethality due to cardiomyopathy (Muller et al., 2003), so its essential physiological and pathophysiological functions were probed in mosaic mice, where Bin1 was partly but not completely ablated throughout the animal (Chang et al., 2007). Through this route, we discovered that Bin1 was a potent driver of IBD (Chang et al., 2012).

In the established dextran sodium sulfate (DSS) model of ulcerative colitis, the most common clinical form of IBD, Bin1 ablation could completely arrest destructive inflammatory processes responsible for disease development, fully preserving colonic integrity and function (Chang et al., 2012). Extending these findings, we found that the therapeutic effect of Bin1 mosaic ablation could be replicated by systemic administration of a cell-penetrating antibody (mAb 99D) that binds to an MYC-binding domain (MBD)-based epitope in Bin1 (Thomas et al., 2016). The effects of mAb 99D were not replicated by a different cell-penetrating antibody (mAb 2F11) that binds to a Bin/Amphiphysin/RVS (BAR) domain-based epitope in Bin1, suggesting that the therapeutic effect of MBD targeting was functionally specific. Notably, the benefit of mAb 99D treatment could be produced in experimental designs for disease prevention (pre-DSS exposure) or disease treatment (post-DSS exposure), suggesting the MBD targeting effect may be useful translationally (Thomas et al., 2016; 2019a).

**FIGURE 2:**
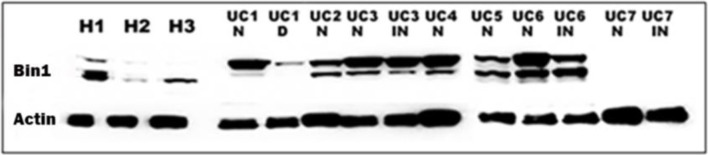
The expression of Bin1 is increased in the colon of UC patients. Western blot of the colon of UC patients (UC 1-7) and healthy subjects (H 1-3) probed with Bin1 antibody. We probed Bin1 levels in Normal (N), Inflamed (IN) and Dysplastic (D) tissues of UC patients.

In functional studies, Bin1 disruption in mouse or human colonic epithelia could preserve barrier function in the face of inflammatory assault. The protective effect of Bin1 disruption was correlated with a restoration of tight junction protein expression patterns that are critical to sustain barrier function (Thomas et al., 2016; Thomas et al., 2019a). Thus, Bin1 promoted IBD by licensing an inflammation-driven degradation of colonic barrier function, with this effect reversed by either Bin1 genetic ablation or Bin1 mAb 99D antibody treatment (Chang et al., 2012; Thomas et al., 2016; Thomas et al., 2019a). Consistent with the concept that Bin1 is an important driver of IBD, an analysis of Bin1 levels in surgically resected human tissues revealed an elevated expression in IBD tissue relative to normal colonic tissue (Figure 2).

## Impact of gut microbiome and enteric nervous system on AD

A dysbiotic gut microbiome along with chronic inflammation and ulceration of the colon are hallmarks of inflammatory bowel disease (IBD). While cause-effect relationships are complex, human population-based cohort studies have demonstrated a significant association between IBD and later development of dementia (Zhang B. et al., 2021; Adewuyi et al., 2022; Aggarwal et al., 2022). A complex gut-brain axis of communications has been documented between the central nervous system (CNS) and the gut-based enteric nervous system (ENS), linking the emotional and cognitive centers of the brain with peripheral intestinal functions.

The gut microbiome has a major impact on ENS structure and function, including to orchestrate bowel movement (Carabotti et al., 2015; Rao and Gershon, 2016), acting as a “second brain” that shares many features with the CNS (Derkinderen et al., 2021). Indeed, ENS dysfunction associated with neuronal loss has been directly linked to aging and dementia (Xia et al., 2022). Constipation is an early sign of AD and Parkinson’s disease (Camacho et al., 2021; Nakase et al., 2022) and regular use of laxatives is associated with a higher risk of dementia (Yang et al., 2023). In contrast, diets rich in fiber that help prevent constipation (Bae, 2014) are associated with lower risks of AD (Martinez-Leo and Segura Campos, 2020; Lei et al., 2021). In other work, evidence has been advanced that gut dysbiosis contributes significantly to risk of AD development and progression, by elevating levels of ENS neuroinflammation, promoting plaque formation, and modifying neurotransmitter production (Zhang Y. et al., 2021).

Gut microorganisms influence ENS and CNS processes bidirectionally via the vagus nerve, modulating the immune system, in part through tryptophan metabolism, and the hypothalamic-pituitary-adrenal axis. We observed that a Bin1-targeted passive immunotherapy (mAb 99D) for IBD altered the status of enteric neurons along with the gut microbiome in association with therapeutic response. Through an ability to directly synthesize certain neurotransmitters and neuroactive metabolites, the gut microbiome may directly affect the ENS (Silva et al., 2020). For example, short-chain fatty acids (SCFA) such as butyric, propionic and acetic acid generated by bacterial fermentation of dietary fibers and resistant starches can potently affect the ENS and CNS (Pascale et al., 2018), acting to stimulate sympathetic nervous system activity, mucosal serotonin release, and memory and learning effects (Silva et al., 2020). Microbiota-generated SCFA also provide a major energy source for enforcing gut barrier function by colon epithelial cells (Parada Venegas et al., 2019). Overall, SCFA production by gut microbiome may be a key contributor to neuro-inflammatory control in the ENS and CNS (Toledo et al., 2022).

In terms of microbiome changes, several studies have correlated age-associated reductions in *Fimicutes,* the major butyrate-producing bacteria in the gut benefiting from a high fiber diet (Parada Venegas et al., 2019), were a key risk factor in AD (Claesson et al., 2011; Burgaletto et al., 2020; Thomas et al., 2022; Chandra et al., 2023). Clinical and murine studies have also suggested gut *Bifidobacterium* as protective against AD, with evidence of improved memory, reduced brain plaque burden, and reduced hippocampal neuro-inflammation (Kobayashi et al., 2017; Xiao et al., 2020; Cao et al., 2021; Kim et al., 2021). A recent study demonstrated that the bacteria *Collinsella, Veillonella, Lachnospira* and *Bacteroides* are risk factors for AD (Cammann et al., 2023).

There is some evidence that tau pathology may arise in the ENS and contribute to the emergence of tau pathology in the CNS (Derkinderen et al., 2021). Phosphorylated tau is expressed in neurons of the human and rodent ENS (Lionnet et al., 2018). Tau pathology in the ENS has been suggested to contribute to tau pathology in the brain, which develops for up to 20 years before overt AD symptoms arise (Braak et al., 2006; Beason-Held et al., 2013). We observed expression of phosphorylated tau in the colon of the PS19 mouse model of tauopathy/AD (Thomas et al., 2019a). A study of human tau in the mouse colon documented loss of enteric neurons due to the accumulation of tau pathology (Xia et al., 2022). A parallel process for α-synuclein accumulation has been suggested in Parkinson’s disease where ENS pathogenicity is clearly coordinated. In the ENS of Parkinson’s patients, α-synuclein overexpression impairs colonic barrier function via caspase-1-inflammasome signaling before brain pathology arises (Yan et al., 2018; Pellegrini et al., 2022). Notably, α-synuclein can be transported into the brain via the vagus nerve (Lei et al., 2021). From these roots, one might hypothesize that tau pathology in the ENS might precede and contribute to future development of AD in the CNS during aging.

## Bin1 monoclonal antibody 99D: a candidate passive immunotherapy to prevent or slow progression of AD

Given evidence implicating Bin1 elevation in phosphorylated tau accumulation and neurofibrillary tangle pathology in AD, we explored whether administration of the Bin1 mAb 99D that produced a preclinical therapeutic benefit in IBD could produce any parallel benefits in model of tauopathy/AD. In cell culture, administration of this cell-penetrating antibody was sufficient to lower levels of endogenous Bin1 and phosphorylated tau, compared to control treatments. Cellular uptake of the antibody was documented by at least two mechanisms involving endosomal proteins and Fc gamma receptors, confirming previous observations in IBD studies (Thomas et al., 2019a). In tau-overexpressing cells, lowering of phosphorylated tau levels by mAb 99D was associated with a specific activation of the native proteasomal machinery and a specific reduction in the level of stress biomarkers expressed by the cells.

An *in vivo* study was conducted in the P301S transgenic mouse model of tauopathy/AD to assess effects of mAb 99D administration on subject survival. We observed that bimonthly dosing over several months extended mouse survival, relative to untreated or murine immunoglobulin control-treated subjects that uniformly expired from disease (Thomas et al., 2019b). While preliminary, these findings encourage further research into the potential therapeutic benefits of this bioactive Bin1 MBD-targeting mAb to lower expression and deposition of phosphorylated tau, as a possible approach to treat AD and/or other tauopathies.

Studies to explore Bin1 as a therapeutic target for passive immunotherapy in AD offer an opportunity to probe emerging concepts in the field as discussed above. Human genetic data provide a rationale to target Bin1 as a risk factor in AD, but its contribution may be direct and/or indirect in terms of mechanistic contributions. Based on evidence of an ability to bind tau and influence its extracellular deposition, Bin1 targeting by mAb 99D may exert effects on tau deposition as a disease driver. Such an effect might not necessarily involve brain penetration; indeed, there is no evidence as yet that mAb 99D enters the brain despite pilot evidence it may limit tauopathy progression in the P301S mouse model. The established benefit of mAb 99D in limiting IBD is intriguing in suggesting the possibility of anti-neuroinflammatory effects in the ENS that might indirectly influence CNS. But would such effects be superior to disease control compared to targeting phosphorylated tau itself? There is certainly strong preclinical evidence in animal models for active or passive immunological strategies to directly attack phosphorylated tau (d’Abramo et al., 2013; Castillo-Carranza et al., 2015; Dai et al., 2015; Asuni et al., 2007; Rajamohamedsait et al., 2017; Corsetti et al., 2020; Bittar et al., 2022). However, different strategies to target a disease driver (tau) versus a disease modifier (Bin1) may be beneficial to explore in different but parallel thrusts.

In future research, it will be important to probe the effects of mAb 99D on tau pathology in the gut ENS of tauopathy/AD models and to determine whether such effects precede and/or affect tau accumulation and AD pathology in the CNS. Looking ahead, mAb 99D may provide a unique probe of Bin1 function in AD, perhaps with the opportunity to illuminate how gut-brain connections might be leveraged to prevent, arrest or reverse AD pathology, especially with regard to potential contributions of neuro-inflammation as a disease modifier as well as phosphorylated tau as a disease driver.

## References

[B1] Adewuyi E. O. O’Brien E. K. Nyholt D. R. Porter T. Laws S. M. (2022). A large-scale genome-wide cross-trait analysis reveals shared genetic architecture between Alzheimer’s disease and gastrointestinal tract disorders. Commun. Biol. 5, 691. 10.1038/s42003-022-03607-2 35851147 PMC9293965

[B2] Aggarwal M. Alkhayyat M. Abou Saleh M. Sarmini M. T. Singh A. Garg R. (2022). Alzheimer disease occurs more frequently in patients with inflammatory bowel disease: Insight from a nationwide study. J. Clin. Gastroenterol. 57, 501–507. 10.1097/MCG.0000000000001714 35470286

[B3] Asuni A. A. Boutajangout A. Quartermain D. Sigurdsson E. M. (2007). Immunotherapy targeting pathological tau conformers in a tangle mouse model reduces brain pathology with associated functional improvements. J. Neurosci. 27 (34), 9115–9129. 10.1523/JNEUROSCI.2361-07.2007 17715348 PMC6672191

[B4] Bae S. H. (2014). Diets for constipation. Pediatr. Gastroenterol. Hepatol. Nutr. 17 (4), 203–208. 10.5223/pghn.2014.17.4.203 25587519 PMC4291444

[B5] Barbier P. Zejneli O. Martinho M. Lasorsa A. Belle V. Smet-Nocca C. (2019). Role of tau as a microtubule-associated protein: Structural and functional aspects. Front. Aging Neurosci. 11, 204. 10.3389/fnagi.2019.00204 31447664 PMC6692637

[B6] Beason-Held L. L. Goh J. O. An Y. Kraut M. A. O'Brien R. J. Ferrucci L. (2013). Changes in brain function occur years before the onset of cognitive impairment. J. Neurosci. 33 (46), 18008–18014. 10.1523/JNEUROSCI.1402-13.2013 24227712 PMC3828456

[B7] Bittar A. Al-Lahham R. Bhatt N. Moore K. Montalbano M. Jerez C. (2022). Passive immunotherapy targeting tau oligomeric strains reverses tauopathy phenotypes in aged human-tau mice in a mouse model-specific manner. J. Alzheimers Dis. 90 (3), 1103–1122. 10.3233/JAD-220518 36189593

[B8] Braak H. Alafuzoff I. Arzberger T. Kretzschmar H. Del Tredici K. (2006). Staging of Alzheimer disease-associated neurofibrillary pathology using paraffin sections and immunocytochemistry. Acta Neuropathol. 112 (4), 389–404. 10.1007/s00401-006-0127-z 16906426 PMC3906709

[B9] Braak H. Braak E. (1991). Neuropathological stageing of Alzheimer-related changes. Acta Neuropathol. 82, 239–259. 10.1007/BF00308809 1759558

[B10] Brion J. P. Couck A. M. Passareiro E. Flament-Durand J. (1985). Neurofibrillary tangles of Alzheimer's disease: An immunohistochemical study. J. Submicrosc. Cytol. 17 (1), 89–96. 3973960

[B11] Burgaletto C. Munafò A. Di Benedetto G. De Francisci C. Caraci F. Di Mauro R. (2020). The immune system on the TRAIL of Alzheimer's disease. J. Neuroinflammation 17 (1), 298. 10.1186/s12974-020-01968-1 33050925 PMC7556967

[B12] Calafate S. Flavin W. Verstreken P. Moechars D. (2016). Loss of Bin1 promotes the propagation of tau pathology. Cell Rep. 17 (4), 931–940. 10.1016/j.celrep.2016.09.063 27760323

[B13] Camacho M. Macleod A. D. Evans J. R. Breen D. P. Cummins G. (2021). Early constipation predicts faster dementia onset in Parkinson’s disease. npj Park. Dis. 7, 45. 10.1038/s41531-021-00191-w PMC8154963 34039994

[B14] Cammann D. Lu Y. Zhang M. L. Cue J. M. Do J. (2023). Genetic correlations between Alzheimer’s disease and gut microbiome genera. Sci. Rep. 13, 5258. 10.1038/s41598-023-31730-5 37002253 PMC10066300

[B15] Cao J. Amakye W. K. Liu X. Ren J. (2021). Bifidobacterium Lactis Probio-M8 regulates gut microbiota to alleviate Alzheimer’s disease in the APP/PS1 mouse model. Eur. J. Nutr. 60, 3757–3769. 10.1007/s00394-021-02543-x 33796919

[B16] Carabotti M. Scirocco A. Maselli M. A. Severi C. (2015). The gut-brain axis: Interactions between enteric microbiota, central and enteric nervous systems. Ann. Gastroenterol. 28 (2), 203–209. 25830558 PMC4367209

[B17] Castillo-Carranza D. L. Guerrero-Muñoz M. J. Sengupta U. Hernandez C. Barrett A. D. Dineley K. (2015). Tau immunotherapy modulates both pathological tau and upstream amyloid pathology in an Alzheimer's disease mouse model. J. Neurosci. 35 (12), 4857–4868. 10.1523/JNEUROSCI.4989-14.2015 25810517 PMC6705372

[B18] Chandra S. Sisodia S. S. Vassar R. J. (2023). The gut microbiome in Alzheimer’s disease: What we know and what remains to be explored. Mol. Neurodegener. 18, 9. 10.1186/s13024-023-00595-7 36721148 PMC9889249

[B19] Chang M. Y. Boulden J. Sutanto-Ward E. DuHadaway J. B. Katz J. B. Wang L. (2007a). Bin1 ablation increases susceptibility to cancer during aging, particularly lung cancer. Cancer Res. 67, 7605–7612. 10.1158/0008-5472.CAN-07-1100 17699764

[B20] Chang M. Y. Boulden J. Sutanto-Ward E. DuHadaway J. B. Soler A. P. Muller A. J. (2007b). Bin1 ablation in mammary gland delays tissue remodeling and drives cancer progression. Cancer Res. 67, 100–107. 10.1158/0008-5472.CAN-06-2742 17210688

[B21] Chang M. Y. Boulden J. Valenzano M. C. Soler A. P. Muller A. J. Mullin J. M. (2012). Bin1 attenuation suppresses experimental colitis by enforcing intestinal barrier function. Dig. Dis. Sci. 57, 1813–1821. 10.1007/s10620-012-2147-y 22526583 PMC3677578

[B22] Chapuis J. Hansmannel F. Gistelinck M. Mounier A. Van Cauwenberghe C. Kolen K. V. (2013). Increased expression of BIN1 mediates Alzheimer genetic risk by modulating tau pathology. Mol. Psychiatry 18 (11), 1225–1234. 10.1038/mp.2013.1 23399914 PMC3807661

[B23] Chung D. C. Roemer S. Petrucelli L. Dickson D. W. (2021). Cellular and pathological heterogeneity of primary tauopathies. Mol. Neurodegener. 16, 57. 10.1186/s13024-021-00476-x 34425874 PMC8381569

[B24] Claesson M. J. Cusack S. O'Sullivan O. Greene-Diniz R. de Weerd H. Flannery E. (2011). Composition, variability, and temporal stability of the intestinal microbiota of the elderly. Proc. Natl. Acad. Sci. U. S. A. 108, 4586–4591. 10.1073/pnas.1000097107 20571116 PMC3063589

[B25] Corsetti V. Borreca A. Latina V. Giacovazzo G. Pignataro A. Krashia P. (2020). Passive immunotherapy for N-truncated tau ameliorates the cognitive deficits in two mouse Alzheimer's disease models. Brain Commun. 2 (1), fcaa039. 10.1093/braincomms/fcaa039 32954296 PMC7425324

[B26] Crotti A. Sait H. R. McAvoy K. M. Estrada K. Ergun A. Szak S. (2019). BIN1 favors the spreading of Tau via extracellular vesicles. Sci. Rep. 9 (1), 9477. 10.1038/s41598-019-45676-0 31263146 PMC6603165

[B27] Cruz-Sanabria F. Bonilla-Vargas K. Estrada K. Mancera O. Vega E. Guerrero E. (2021). Analysis of cognitive performance and polymorphisms of SORL1, PVRL2, CR1, TOMM40, APOE, PICALM, GWAS_14q, CLU, and BIN1 in patients with mild cognitive impairment and cognitively healthy controls. Neurol. Engl. Ed. 36 (9), 681–691. 10.1016/j.nrleng.2018.07.012 34752346

[B28] Cummings J. Aisen P. S. DuBois B. Frölich L. Jack C. R. Jones R. W. (2016). Drug development in Alzheimer's disease: The path to 2025. Alzheimers Res. Ther. 39. 10.1186/s13195-016-0207-9 PMC5028936 27646601

[B29] Cummings J. L. (2004). Alzheimer's disease. N. Engl. J. Med. 351 (1), 56–67. 10.1056/nejmra040223 15229308

[B30] d'Errico P. Meyer-Luehmann M. (2020). Mechanisms of pathogenic tau and Aβ protein spreading in Alzheimer's disease. Front. Aging Neurosci. 12, 265. 10.3389/fnagi.2020.00265 33061903 PMC7481386

[B31] d’Abramo C. Acker C. M. Jimenez H. T. Davies P. (2013). Tau passive immunotherapy in mutant P301L mice: Antibody affinity versus specificity. PLoS ONE 8 (4), e62402. 10.1371/journal.pone.0062402 23638068 PMC3639259

[B32] Dai C. L. Chen X. Kazim S. F. Liu F. Gong C. X. Grundke-Iqbal I. (2015). Passive immunization targeting the N-terminal projection domain of tau decreases tau pathology and improves cognition in a transgenic mouse model of Alzheimer disease and tauopathies. J. Neural Transm. (Vienna) 122 (4), 607–617. 10.1007/s00702-014-1315-y 25233799

[B33] Daudin R. Marechal D. Wang Q. Abe Y. Bourg N. Sartori M. (2018). BIN1 genetic risk factor for Alzheimer is sufficient to induce early structural tract alterations in entorhinal cortex-dentate gyrus pathway and related hippocampal multi-scale impairments. bioRxiv. 437228.

[B34] Derkinderen P. Rolli-Derkinderen M. Chapelet G. Neunlist M. Noble W. (2021). Tau in the gut, does it really matter? J. Neurochem. 158 (2), 94–104. 10.1111/jnc.15320 33569813

[B35] Dräger N. M. Nachman E. Winterhoff M. Brühmann S. Shah P. Katsinelos T. (2017). Bin1 directly remodels actin dynamics through its BAR domain. EMBO Rep. 18 (11), 2051–2066. 10.15252/embr.201744137 28893863 PMC5666605

[B36] Fulop T. Witkowski J. M. Bourgade K. Khalil A. Zerif E. Larbi A. (2018). Can an infection hypothesis explain the beta amyloid hypothesis of Alzheimer's disease? Front. Aging Neurosci. 10, 224. 10.3389/fnagi.2018.00224 30087609 PMC6066504

[B37] Glennon E. B. Lau D. H. Gabriele R. M. C. Taylor M. F. Troakes C. Opie-Martin S. (2020). Bridging Integrator-1 protein loss in Alzheimer's disease promotes synaptic tau accumulation and disrupts tau release. Brain Commun. 2 (1), fcaa011. 10.1093/braincomms/fcaa011 32500121 PMC7272218

[B38] Grundke-Iqbal I. Iqbal K. Tung Y. C. Quinlan M. Wisniewski H. M. Binder L. I. (1986). Abnormal phosphorylation of the microtubule-associated protein tau (tau) in Alzheimer cytoskeletal pathology. Proc. Natl. Acad. Sci. U. S. A. 83, 4913–4917. 10.1073/pnas.83.13.4913 3088567 PMC323854

[B39] Hippius H. Neundörfer G. (2003). The discovery of Alzheimer's disease. Dialogues Clin. Neurosci. 5 (1), 101–108. 10.31887/DCNS.2003.5.1/hhippius 22034141 PMC3181715

[B40] Holler C. J. Davis P. R. Beckett T. L. Platt T. L. Webb R. L. Head E. (2014). Bridging integrator 1 (BIN1) protein expression increases in the Alzheimer's disease brain and correlates with neurofibrillary tangle pathology. J. Alzheimers Dis. 42 (4), 1221–1227. 10.3233/JAD-132450 25024306 PMC4198456

[B41] Kim H. Kim S. Park S. J. Park G. Shin H. Park M. S. (2021). Administration of Bifidobacterium bifidum BGN4 and Bifidobacterium longum BORI improves cognitive and memory function in the mouse model of Alzheimer's disease. Front. Aging Neurosci. 13, 709091. 10.3389/fnagi.2021.709091 34421576 PMC8378450

[B42] Kobayashi Y. Sugahara H. Shimada K. Mitsuyama E. Kuhara T. Yasuoka A. (2017). Therapeutic potential of Bifidobacterium breve strain A1 for preventing cognitive impairment in Alzheimer’s disease. Sci. Rep. 7, 13510. 10.1038/s41598-017-13368-2 29044140 PMC5647431

[B43] Kumar D. K. Choi S. H. Washicosky K. J. Eimer W. A. Tucker S. Ghofrani J. (2016). Amyloid-β peptide protects against microbial infection in mouse and worm models of Alzheimer's disease. Sci. Transl. Med. 8 (340), 340ra72. 340ra72. 10.1126/scitranslmed.aaf1059 PMC5505565 27225182

[B44] Kunkle B. W. Grenier-Boley B. Sims R. Bis J. C. Damotte V. Naj A. C. (2019). Genetic meta-analysis of diagnosed Alzheimer's disease identifies new risk loci and implicates Aβ, tau, immunity and lipid processing. Nat. Genet. 51 (3), 414–430. 10.1038/s41588-019-0358-2 30820047 PMC6463297

[B45] Laury-Kleintop L. D. Mulgrew J. R. Heletz I. Nedelcoviciu R. A. Chang M. Y. Harris D. M. (2015). Cardiac-specific disruption of Bin1 in mice enables a model of stress- and age-associated dilated cardiomyopathy. J. Cell. Biochem. 116, 2541–2551. 10.1002/jcb.25198 25939245

[B46] Lei Q. Wu T. Wu J. Hu X. Guan Y. Wang Y. (2021). Roles of α-synuclein in gastrointestinal microbiome dysbiosis-related Parkinson's disease progression (Review). Mol. Med. Rep. 24 (4), 734. 10.3892/mmr.2021.12374 34414447 PMC8404091

[B47] Lionnet A. Wade M. A. Corbillé A. G. Prigent A. Paillusson S. Tasselli M. (2018). Characterisation of tau in the human and rodent enteric nervous system under physiological conditions and in tauopathy. Acta Neuropathol. Commun. 6 (1), 65. 10.1186/s40478-018-0568-3 30037345 PMC6055332

[B48] Liu F. Iqbal K. Grundke-Iqbal I. Hart G. W. Gong C. X. (2004). O-GlcNAcylation regulates phosphorylation of tau: A mechanism involved in Alzheimer's disease. Proc. Natl. Acad. Sci. U. S. A. 101, 10804–10809. 10.1073/pnas.0400348101 15249677 PMC490015

[B49] Malki I. Cantrelle F. X. Sottejeau Y. Lippens G. Lambert J. C. Landrieu I. (2017). Regulation of the interaction between the neuronal BIN1 isoform 1 and Tau proteins - role of the SH3 domain. FEBS J. 284, 3218–3229. 10.1111/febs.14185 28755476

[B50] Márquez F. Yassa M. A. (2019). Neuroimaging biomarkers for Alzheimer’s disease. Mol. Neurodegener. 14, 21. 10.1186/s13024-019-0325-5 31174557 PMC6555939

[B51] Martínez Leo E. E. Segura Campos M. R. (2020). Effect of ultra-processed diet on gut microbiota and thus its role in neurodegenerative diseases. Nutrition 71, 110609. 10.1016/j.nut.2019.110609 31837645

[B52] Medeiros R. Baglietto-Vargas D. LaFerla F. M. (2011). The role of tau in Alzheimer's disease and related disorders. CNS Neurosci. Ther. 17, 514–524. 10.1111/j.1755-5949.2010.00177.x 20553310 PMC4072215

[B53] Miyagawa T. Ebinuma I. Morohashi Y. Hori Y. Young Chang M. Hattori H. (2016). BIN1 regulates BACE1 intracellular trafficking and amyloid-β production. Hum. Mol. Genet. 25 (14), 2948–2958. 10.1093/hmg/ddw146 27179792

[B54] Miyagawa T. Morohashi Y. Hori Y. Tsuji S. Iwatsubo T. Chang M. Y. (2016). BIN1 regulates BACE1 intracellular trafficking and amyloid-β production. Hum. Mol. Genet. 25, 2948–2958. 10.1093/hmg/ddw146 27179792

[B55] Mudher A. Colin M. Dujardin S. Medina M. Dewachter I. Alavi Naini S. M. (2017). What is the evidence that tau pathology spreads through prion-like propagation? Acta Neuropathol. Commun. 5 (1), 99. 10.1186/s40478-017-0488-7 29258615 PMC5735872

[B56] Muller A. J. Baker J. F. DuHadaway J. B. Ge K. Farmer G. Meade R. (2003). Targeted disruption of the murine Bin1/Amphiphysin II gene does not disable endocytosis but results in embryonic cardiomyopathy with aberrant myofibril formation. Mol. Cell. Biol. 23, 4295–4306. 10.1128/mcb.23.12.4295-4306.2003 12773571 PMC156129

[B92] Muller A. J. DuHadaway J. B. Donover P. S. Sutanto-Ward E. Prendergast G. C. (2004). Targeted deletion of the suppressor gene bin1/amphiphysin2 accentuates the neoplastic character of transformed mouse fibroblasts. Cancer Biol. Ther. 3, 1236–1242. 10.4161/cbt.3.12.1232 15611650

[B93] Muller A. J. Mondal A. Dey S. Prendergast G. C. (2023). IDO1 and inflammatory neovascularization: bringing new blood to tumor-promoting inflammation. Front. Oncol. 13, 1165298. 10.3389/fonc.2023.1165298 37182174 PMC10172587

[B57] Murphy M. P. LeVine H. 3rd (2010). Alzheimer's disease and the amyloid-beta peptide. J. Alzheimers Dis. 19 (1), 311–323. 10.3233/JAD-2010-1221 20061647 PMC2813509

[B58] Nakase T. Tatewaki Y. Thyreau B. Mutoh T. Tomita N. Yamamoto S. (2022). Impact of constipation on progression of Alzheimer's disease: A retrospective study. CNS Neurosci. Ther. 28, 1964–1973. 10.1111/cns.13940 35934956 PMC9627372

[B59] Parada Venegas D. De la Fuente M. K. Landskron G. González M. J. Quera R. Dijkstra G. (2019). Short chain fatty acids (SCFAs)-Mediated gut epithelial and immune regulation and its relevance for inflammatory bowel diseases. Front. Immunol. 10, 277. 10.3389/fimmu.2019.00277 30915065 PMC6421268

[B60] Pascale A. Marchesi N. Marelli C. Coppola A. Luzi L. Govoni S. (2018). Microbiota and metabolic diseases. Endocrine 61, 357–371. 10.1007/s12020-018-1605-5 29721802

[B61] Pellegrini C. D’Antongiovanni V. Miraglia F. Rota L. Benvenuti L. Di Salvo C. (2022). Enteric α-synuclein impairs intestinal epithelial barrier through caspase-1-inflammasome signaling in Parkinson’s disease before brain pathology. Park. Dis. 8, 9. 10.1038/s41531-021-00263-x PMC8755783 35022395

[B62] Perdigão C. Barata M. A. Burrinha T. Guimas Almeida C. (2021). Alzheimer's disease BIN1 coding variants increase intracellular Aβ levels by interfering with BACE1 recycling. J. Biol. Chem. 297 (3), 101056. 10.1016/j.jbc.2021.101056 34375641 PMC8413894

[B63] Pooler A. M. Phillips E. C. Lau D. H. Noble W. Hanger D. P. (2013). Physiological release of endogenous tau is stimulated by neuronal activity. EMBO Rep. 14, 389–394. 10.1038/embor.2013.15 23412472 PMC3615658

[B64] Prendergast G. C. Muller A. J. Ramalingam A. Chang M. Y. (2009). BAR the door: Cancer suppression by amphiphysin-like genes. Biochim. Biophys. Acta 1795, 25–36. 10.1016/j.bbcan.2008.09.001 18930786 PMC2874822

[B65] Qin W. Ho L. Wang J. Peskind E. Pasinetti G. M. (2009). S100A7, a novel Alzheimer's disease biomarker with non-amyloidogenic alpha-secretase activity acts via selective promotion of ADAM-10. PLoS ONE 4, e4183. 10.1371/journal.pone.0004183 19159013 PMC2613557

[B66] Rajamohamedsait H. Rasool S. Rajamohamedsait W. Lin Y. Sigurdsson E. M. (2017). Prophylactic active tau immunization leads to sustained reduction in both tau and amyloid-β pathologies in 3xTg mice. Sci. Rep. 7, 17034. 10.1038/s41598-017-17313-1 29213096 PMC5719023

[B67] Rao M. Gershon M. D. (2016). The bowel and beyond: The enteric nervous system in neurological disorders. Nat. Rev. Gastroenterol. Hepatol. 13, 517–528. 10.1038/nrgastro.2016.107 27435372 PMC5005185

[B68] Saha P. Sen N. (2019). Tauopathy: A common mechanism for neurodegeneration and brain aging. Mech. Ageing Dev. 178, 72–79. 10.1016/j.mad.2019.01.007 30668956 PMC6377302

[B69] Sartori M. Mendes T. Desai S. Lasorsa A. Herledan A. Malmanche N. (2019). BIN1 recovers tauopathy-induced long-term memory deficits in mice and interacts with Tau through Thr(348) phosphorylation. Acta Neuropathol. 138, 631–652. 10.1007/s00401-019-02017-9 31065832 PMC6778065

[B70] Seshadri S. Fitzpatrick A. L. Ikram M. A. DeStefano A. L. Gudnason V. Boada M. (2010). Genome-wide analysis of genetic loci associated with Alzheimer disease. JAMA 303, 1832–1840. 10.1001/jama.2010.574 20460622 PMC2989531

[B71] Silva Y. P. Bernardi A. Frozza R. L. (2020). The role of short-chain fatty acids from gut microbiota in gut-brain communication. Front. Endocrinol. (Lausanne) 11, 25. 10.3389/fendo.2020.00025 32082260 PMC7005631

[B72] Sottejeau Y. Bretteville A. Cantrelle F. X. Malmanche N. Demiaute F. Mendes T. (2015). Tau phosphorylation regulates the interaction between BIN1’s SH3 domain and Tau’s proline-rich domain. Acta Neuropathol. Commun. 3, 58. 10.1186/s40478-015-0237-8 26395440 PMC4580349

[B73] Taga M. Petyuk V. A. White C. Marsh G. Ma Y. Klein H. U. (2020). BIN1 protein isoforms are differentially expressed in astrocytes, neurons, and microglia: Neuronal and astrocyte BIN1 are implicated in tau pathology. Mol. Neurodegener. 15, 44. 10.1186/s13024-020-00387-3 32727516 PMC7389646

[B74] Tan M. S. Yu J. T. Tam L. (2013). Bridging integrator 1 (BIN1): Form, function, and Alzheimer’s disease. Trends Mol. Med. 19, 594–603. 10.1016/j.molmed.2013.06.004 23871436

[B75] Therriault J. Zimmer E. R. Benedet A. L. Pascoal T. A. Gauthier S. Rosa-Neto P. (2022a). Staging of Alzheimer's disease: Past, present, and future perspectives. Trends Mol. Med. 28, 726–741. 10.1016/j.molmed.2022.05.008 35717526

[B76] Therriault J. Pascoal T. A. Lussier F. Z. Tissot C. Chamoun M. Bezgin G. (2022). Biomarker modeling of Alzheimer’s disease using PET-based Braak staging. Nat. Aging 2, 526–535. 10.1038/s43587-022-00204-0 37118445 PMC10154209

[B77] Thomas S. Hoxha K. Alexander W. Gilligan J. Dilbarova R. Whittaker K. (2019a). Intestinal barrier tightening by a cell-penetrating antibody to Bin1, a candidate target for immunotherapy of ulcerative colitis. J. Cell Biochem. 120, 4225–4237. 10.1002/jcb.27716 30269357

[B78] Thomas S. Hoxha K. Tran A. Prendergast G. C. (2019b). Bin1 antibody lowers the expression of phosphorylated Tau in Alzheimer's disease. J. Cell Biochem. 120, 18320–18331. 10.1002/jcb.29142 31211444

[B79] Thomas S. Mercado J. M. DuHadaway J. DiGuilio K. Mullin J. M. Prendergast G. C. (2016). Novel colitis immunotherapy targets Bin1 and improves colon cell barrier function. Dig. Dis. Sci. 61, 423–432. 10.1007/s10620-015-3804-8 26195312

[B80] Thomas S. Mercogliano G. Prendergast G. C. (2022). Bin1 targeted immunotherapy alters the status of the enteric neurons and the microbiome during ulcerative colitis treatment. PLoS ONE 17, e0276910. 10.1371/journal.pone.0276910 36322599 PMC9629549

[B81] Toledo A. R. L. Monroy G. R. Salazar F. E. Lee J. Y. Jain S. Yadav H. (2022). Gut-brain Axis as a pathological and therapeutic target for neurodegenerative disorders. Int. J. Mol. Sci. 23, 1184. 10.3390/ijms23031184 35163103 PMC8834995

[B82] Uberti D. Cantarella G. Facchetti F. Cafici A. Grasso G. Bernardini R. (2004). TRAIL is expressed in the brain cells of Alzheimer's disease patients. Neuroreport 15 (4), 579–581. 10.1097/00001756-200403220-00002 15094456

[B83] Usardi A. Pooler A. M. Seereeram A. Reynolds C. H. Derkinderen P. Anderton B. (2011). Tyrosine phosphorylation of tau regulates its interactions with Fyn SH2 domains, but not SH3 domains, altering the cellular localization of tau. FEBS J. 278, 2927–2937. 10.1111/j.1742-4658.2011.08218.x 21692989

[B84] Wang H. F. Wan Y. Hao X. K. Cao L. Zhu X. C. Jiang T. (2016). Neuroimaging initiative Alzheimer’s disease: Bridging integrator 1 (BIN1) genotypes mediate Alzheimer's disease risk by altering neuronal degeneration. J. Alzheimers Dis. 52, 179–190. 10.3233/JAD-150972 27003210

[B85] Wood J. Zinsmeister P. (1989). Immunohistochemical evidence for reorganization of tau in the plaques and tangles in Alzheimer's disease. Histochem J. 21, 659–662. 10.1007/BF01002486 2511165

[B86] Xia Y. Prokop S. Bell B. M. Gorion K. M. M. Croft C. L. Nasif L. (2022). Pathogenic tau recruits wild-type tau into brain inclusions and induces gut degeneration in transgenic SPAM mice. Commun. Biol. 5, 446. 10.1038/s42003-022-03373-1 35550593 PMC9098443

[B87] Xiao J. Katsumata N. Bernier F. Ohno K. Yamauchi Y. Odamaki T. (2020). Probiotic Bifidobacterium breve in improving cognitive functions of older adults with suspected mild cognitive impairment: A randomized, double-blind, placebo-controlled trial. J. Alzheimers Dis. 77, 139–147. 10.3233/JAD-200488 32623402 PMC7592675

[B88] Yan F. Chen Y. Li M. Wang Y. Zhang W. Chen X. (2018). Gastrointestinal nervous system α-synuclein as a potential biomarker of Parkinson disease. Med. Baltim. 97, e11337. 10.1097/MD.0000000000011337 PMC6076112 29995769

[B89] Yang Z. Wei C. Li X. Yuan J. Gao X. Li B. (2023). Association between regular laxative use and incident dementia in UK biobank participants. Neurology 10, e1702–e1711. (in press). 10.1212/WNL.0000000000207081 PMC10115504 36813729

[B90] Zhang B. Wang H. E. Bai Y. M. Tsai S. J. Su T. P. Chen T. J. (2021a). Inflammatory bowel disease is associated with higher dementia risk: A nationwide longitudinal study. Gut 70, 85–91. 10.1136/gutjnl-2020-320789 32576641

[B91] Zhang Y. Geng R. Tu Q. (2021b). Gut microbial involvement in Alzheimer's disease pathogenesis. Aging (Albany NY) 13, 13359–13371. 10.18632/aging.202994 33971619 PMC8148443

